# Resin cementation of zirconia ceramics with different bonding agents

**DOI:** 10.1080/13102818.2014.996606

**Published:** 2015-01-09

**Authors:** Merve Çakırbay Tanış, Canan Akay, Duygu Karakış

**Affiliations:** ^a^ Private Dental Specialist, Prosthodontists, Ankara, Turkey; ^b^ Department of Prosthetic Dentistry, Faculty of Dentistry, Gazi University, Ankara, Turkey

**Keywords:** zirconia, resin cement, MDP monomer, primer agents

## Abstract

The aim of this study was to evaluate the effects of sandblasting and different chemical bonding agents on shear bond strength of zirconia and conventional resin cement. In this study, 35 zirconia specimens were treated as follows: Group I: control; Group II: sandblasting; Group III: sandblasting + Monobond S; Group IV: sandblasting + Monobond Plus; Group V: sandblasting + Z-Prime Plus. The specimens in each group were bonded with conventional composite resin cement Variolink II. After cementation, specimens were stored in distilled water (at 37 °C) for 24 h and shear test was performed. The highest shear bond strength values were observed in Groups IV and V. The lowest shear bond strength values were observed in Group I. Using 10-methacryloyloxy-decyl dihydrogenphosphate monomer-containing priming agents, e.g. Monobond Plus and Z-PRIME Plus, combined with sandblasting can be an effective method for resin bonding of zirconia restorations.

## Introduction

The use of all-ceramic dental restorations has increased in recent years due to their superior aesthetic appearance and metal-free structure.[1,2] Zirconium has been introduced for dental use as a core material for conventional and resin-bonded fixed partial dentures and complete coverage crowns due to their improved mechanical properties in comparison to more conventional alumina or lithium disilicate-based ceramics.[1,3] Although improved mechanical properties are important for the long-term performance of a ceramic material, the clinical success of fixed ceramic prostheses seems to be strongly dependent on the cementation procedure.[4] Zirconia restorations can be bonded to teeth structures by conventional cements and resin cements;[5] however, resin cements are more preferred because they have the advantages of marginal seal, good retention and improvement of fracture resistance of ceramic materials.[6]

It is generally considered that conventional methods of adhesive cementation, which include prior acid etching of the ceramic surface with hydrofluoric acid and further silanation, are not efficient for zirconia ceramics because of their lack of silica and glass phase.[4] Silane molecules react with water to form silanol groups (--Si–OH) from methoxy groups (--Si–O–CH_3_) and the silanol groups react with the silica surface to form a siloxane (--Si–O–Si–O--) network.[7,8] Therefore, in order to achieve acceptable bonding between silica-free zirconia and resin cement, alternative methods are required.[9]

Many surface roughening methods, such as sandblasting, laser etching, selective infiltration etching and nano-structured alumina coating, have been developed for achieving micromechanical interlocking between zirconia and resin cement. Sandblasting is the most preferred surface-roughening method for zirconia ceramics.[10,11] This method increases the surface energy and wettability but can weaken the ceramic by creating microcracks on the zirconia surface. However, it has been shown that resin luting agents heal the minor surface flaws created by sandblasting and strengthen the ceramic.[11]

Mechanical adhesion achieved by surface roughening is not enough to provide a durable bond between zirconia and resin cement.[12] That is why, adhesive strategies combining surface roughening procedures and chemical bonding, which involve use of various primers and resin cements have been developed.[13] It has been shown that the use of primers or resin cements containing 10-methacryloyloxy-decyl dihydrogenphosphate (MDP) monomer following sandblasting increases the bond strength of zirconia.[14,15] MDP is an acidic phosphate monomer, which is originally designed to bond to metal oxides and its use has been extended to zirconia.[16] It has affinity to metal oxides and zirconia surface is easily covered with a passive oxide layer, which makes zirconia similar to metal, therefore it can be used for increasing the bond strength between zirconia and resin cement.[17,18]

In recent years, manufacturers have developed several bonding agents including different adhesive monomers. The wide variety of available primers makes it difficult for clinicians to choose the correct system for specific clinical situations.[19] There are few studies that report primer comparisons. The purpose of this study was to evaluate the effects of different combinations of mechanical and chemical surface pre-treatments on shear bond strength of zirconia ceramics with a conventional resin cement.

## Materials and methods

### Specimens

Thirty-five (13 mm × 7.5 mm × 2.5 mm) specimens were milled from pre-sintered zirconia blocks (ICE Zirkon, Zirkonzahn, Bruneck, Italy) by using Zirkograph 025 ECO (Zirkonzahn, Bruneck, Italy). The specimens were sintered at 1500 °C for 8 h in a high-temperature sintering furnace for zirconia (Zirkonofen 600/V2, Zirkonzahn, Bruneck, Italy). The final dimensions of the specimens were 10 mm ± 0.4 mm × 5 mm ± 0.4 mm × 2 mm ± 0.3 mm following 20% volumetric shrinkage associated with the sintering. Each specimen was embedded in an autopolymerizing acrylic resin block (Meliodent; Heraeus Kulzer, Armonk, NY) and ground-finished with 600-, 800-, 1000- and 1200-grit silicon carbide abrasives (3M ESPE, St. Paul, MN) under running water on a polishing machine (Metkon Gripo 2V, Bursa, Turkey). All specimens were ultrasonically cleaned in distilled water for 15 min before application of surface treatments and then air dried.

### Surface treatment

Specimens were divided into five groups according to the surface treatment performed:

Group I: Control.
Group II: Specimens in this group were sandblasted with 50 µm Al_2_O_3_ particles (BEGO Korox, Bremen, Germany) from a distance of 10 mm perpendicular to the specimen surface at a pressure of 2.5 bar for 15 s.
Group III: Specimens in this group were sandblasted with 50 µm Al_2_O_3_ particles, following the same procedure as that in Group II. Then, ceramic surfaces were coated with Monobond S (Ivoclar Vivadent, Schaan, Liechtenstein) and allowed to air dry for 5 min.
Group IV: Specimens in this group were sandblasted with 50 µm Al_2_O_3_ particles, following the same procedure as that in Group II. Then, ceramic surfaces were coated with Monobond Plus (Ivoclar Vivadent, Schaan, Liechtenstein) and allowed to air dry for 5 min.
Group V: Specimens in this group were sandblasted with 50 µm Al_2_O_3_ particles, following the same procedure as that in Group II. Then, ceramic surfaces were coated with Z-PRIME Plus (Bisco, Schaumburg, IL, USA) and allowed to air dry for 5 min. Experimental materials and their characteristics are shown in Table 1.

**Table 1.** Experimental materials and their characteristics.

Material	Composition	Manufacturer
Zirconia	ZrO_3_; specifications: Y_2_O_3_ 4–6%, Al_2_O_3_ 1%, SiO_2_ max. 0.02%, Fe_2_O_3_ max. 0.01%, Na_2_O max. 0.04%	ZirkonZahn, Bruneck, Italy
Monobond S	Ethanol, [3-(methacryloyloxy)propyl]trimethoxysilane	Ivocalar Vivadent, Schaan, Liechtenstein
Monobond Plus	Ethanol, 3-(trimethoxysilyl)propyl methacrylate, methacrylated phosphoric acid ester	Ivocalar Vivadent, Schaan, Liechtenstein
Z-PRIME Plus	BPDM, HEMA, ethanol, MDP	Bisco Inc., Schaumburg, IL
Variolink II Base	Bis-GMA, urethane dimethacrylate, triethyleneglycol dimethacrylate	Ivoclar Vivadent, Schaa, Liechtenstein
Variolink II Catalyst	Bis-GMA, urethane dimethacrylate,triethyleneglycol dimethacrylate, dibenzoyl peroxide	Ivoclar Vivadent, Schaan, Liechtenstein

### Experimental procedure

After application of surface treatments, conventional resin cement Variolink II (Ivoclar, Vivadent AG, Schaan Liechtenstein) was applied on the surface of the specimens. Adhesion procedures were performed according to the manufacturer's recommendations.

Plexiglas tubes were placed in the centre of the specimens and resin cements were filled in the tubes with the help of a hand instrument. Then, resin cements were light cured from two opposite sides for 40 s (BlueLEX LD-105, Monitex Industrial Co., Taipei, Taiwan).

After the cementation procedure, specimens were stored in distilled water at 37 °C for 24 h. Following water storage, shear bond strength test was performed at a crosshead speed of 1 mm/min in a universal test machine (Lloyd-LRX; Lloyd Instruments, Fareham, UK).

### Statistical analysis

All data were analysed using Statistical Package for the Social Sciences, version 16.0 (SPSS Inc., Chicago, IL, USA). One-way analysis of variance and Tukey's *post hoc* tests were performed to evaluate the shear bond strength of tested chemical and mechanical surface-conditioning methods. *P* < 0.05 was considered statistically significant.

## Results and discussion

This study evaluated effects of sandblasting and three different bonding agents on shear bond strength of zirconia and conventional resin cement Variolink II. Shear bond strength is the most common test method used for evaluating bond strength of zirconia because no additional process is required once the bonding procedure is completed.[20]

The mean shear bond strength achieved by each experimental protocol is shown in Table 2. All the tested surface treatment methods showed statistically higher shear bond strength values than that in the control group (*P* < 0.05). The improvement of resin bonding of zirconia observed following sandblasting compared to the control group is in agreement with previous studies [10,21,22]. However, the improvement achieved by sandblasting was lower than that shown by the other tested methods. The results in the group treated with sandblasting only were statistically significantly different from the values obtained in all other protocols except for Monobond S (Table 2).

**Table 2.** Shear bond strength test mean values.

	Mean (MPa)	Standard deviation
Control	8.52^a,b,c^	1.19
Sandblasting	11.34^d,e^	2.07
Sandblasting + Monobond S	12.42^a,f^	0.62
Sandblasting + Monobond Plus	19.27^b,d,f,g^	1.79
Sandblasting + Z-Prime Plus	15.25^c,e,g^	1.20

Note. Same superscript letters indicate statistically significance.

### Sandblasting

Sandblasting is known to form surface roughness and irregularities and to increase the surface area and wettability, thus allowing resin cement to flow in to the surface.[16,23] Previous studies have demonstrated that sandblasting affects the crown retention regardless of the cement used.[24] Although sandblasting improves bonding, it can affect the mechanical properties of zirconia [24] due to phase transformation which can cause fatigue in the material structure [25]. However, there are also reports that sandblasting strengthens the mechanical characteristics of zirconia.[14,26,27]

### Priming agents

Most studies report that priming agents are effective when applied with sandblasting.[4,10,17,28–30] Therefore, several priming agents were applied after sandblasting in this study. Surface activation and the cleaning effect of sandblasting are needed for chemical bonding of zirconia.[30] Kitayama et al. [23] reported that the increase of bond strength when a primer agent is used with sandblasting may be due to a rewetting effect on sandblasted zirconia.

#### Silane coupling agent

In this study, the use of the silane coupling agent Monobond S [which is composed of 3-(methacryloyloxy)propyltrimethoxysilane, ethanol and water] combined with sandblasting did not improve significantly (*P* > 0.05) the bond strength between zirconia resin cement when compared with sandblasting only (Table 2). This result suggests that silanes are not the effective agents for improving resin bonding of zirconia. This suggestion is in agreement with the study of Atsu et al. [1], who found that sandblasting followed by application of a silane coupling agent (Clearfil Porcelain Bond Activator) did not statistically significantly improve the bond strength between zirconia and resin cement compared to sandblasting only. However, silanes can be effective when the zirconia surface is coated with silica.[31]

#### Phosphate-monomer-containing agents

Zirconium oxide can react with phosphate ester monomers, which is why MDP-containing primers or resin cements can improve zirconia resin cement bonding.[5] There are several studies reporting that phosphate-monomer-containing primer agents improve resin bond strength of sandblasted zirconia.[11,23,32–34] The results from our study are consistent with this opinion.

In our study, the priming agent Monobond Plus, which contains 3-(trimethoxysilyl)propyl methacrylate (3-MPS) monomer, sulphide methacrylate and methacrylated phosphoric acid ester, resulted in the highest bond strengths (Table 2). This is in agreement with Attia et al. [35], who reported that the use of Monobond Plus combined with sandblasting or tribochemical silica coating enhances durable resin bonding to zirconia ceramic after 30 days water storage and thermal cycling. Similarly, there are several other studies reporting that Monobond Plus increases the bond strength when combined with sandblasting.[6,36]

Application of the MDP-containing priming agent Z-PRIME Plus with sandblasting improved the bond strength of zirconia resin cement compared to only sandblasting or the control group (Table 2). Similarly, Magne et al. [37] reported that use of Z-PRIME Plus increases shear bond strength of zirconia with different resin based luting agents. Zandsparsa et al. [10] and Shin et al. [13] found that use of Z-PRIME Plus with sandblasting improved bond strength of zirconia compared to only sandblasting.

Thus, both phosphate-monomer-containing priming agents, Monobond Plus and Z-PRIME Plus, significantly increased the bond strength of zirconia with resin cement in our experimental design. This suggests that the use of Monobond Plus or Z-PRIME Plus following sandblasting could be considered an effective method for achieving resin bonding of zirconia. Use of ceramic primers, which chemically bond with the metal oxides at zirconia ceramic surface provides initial high bond strength to zirconia.[38] This can be explained by chemical bonding of adhesive monomers to metal oxides through van der Waals forces or hydrogen bonds at the resin zirconia interface.[5]

When the two phosphate-monomer-containing primers used in this study were compared, Monobond Plus showed statistically significantly higher bond strength than Z-PRIME Plus (*P* < 0.05). This result is consistent with the study of Amaral et al. [19], who compared the effects of Z-PRIME Plus and Monobond Plus on polished and sandblasted zirconia surface and demonstrated that application of Monobond Plus yields higher bond strength than Z-PRIME Plus. This can be explained with the composition of Monobond Plus. It contains both an MDP monomer and a silane monomer 3-MPS, which is commonly used in dentistry. There are several studies reporting that application of MDP-containing bonding/silane coupling agent mixtures increases bond strength between sandblasted zirconia and resin cement.[1,11,20,32]

### Limitations

This study used the shear bond strength test because of its easy application procedure. This test method, however, has the disadvantage of inhomogeneous stress distribution.[18,20] Although microtensile test method is recommended for evaluating bond strength of zirconia resin cement [39], Magne et al. [37] demonstrated that shear bond strength test and microtensile test methods give similar results on high-strength ceramics. This study also used static loading instead of dynamic loading. Artificial aging methods were not performed. This may be considered one of the limitations of this study. Studies including thermal cycling and long-term water storage using different priming agents are needed to evaluate bond strength between zirconia and resin luting agents. Another limitation of this study is that only one type of cement was used and the results cannot be generalized to other cements. Within the limitations of this study, it can be suggested that application of priming agents including phosphoric ester monomers is an effective method for improving bond strength between zirconia and the conventional resin cement Variolink II.

## Conclusions

Within the limitations of this *in vitro* study, the obtained results suggest that use of adhesive phosphate-monomer-containing bonding agents following sandblasting is an effective factor for achieving resin cementation of zirconia ceramics. Studies including an aging procedure and different types of cements are needed to more precisely evaluate the bond strength between zirconia and resin luting agents.

## Acknowledgements

This study was presented at the European Prosthodontic Association Congress 2014, Istanbul, Turkey.

## Disclosure statement

No potential conflict of interest was reported by the authors.
